# Impact of Host Plant Species and Whitefly Species on Feeding Behavior of *Bemisia tabaci*

**DOI:** 10.3389/fpls.2019.00001

**Published:** 2019-01-22

**Authors:** Milan Milenovic, Everlyne Nafula Wosula, Carmelo Rapisarda, James Peter Legg

**Affiliations:** ^1^Dipartimento di Agricoltura, Alimentazione e Ambiente, University of Catania, Catania, Italy; ^2^Department of Plant and Environmental Sciences, University of Copenhagen, Copenhagen, Denmark; ^3^International Institute of Tropical Agriculture, Dar es Salaam, Tanzania

**Keywords:** cassava, EPG, whitefly, sweet potato, *Bemisia tabaci*, feeding behavior

## Abstract

Whiteflies of the *Bemisia tabaci* species complex are economically important pests of cassava. In Africa, they cause greatest damage through vectoring viruses responsible for cassava mosaic disease and cassava brown streak disease. Several cryptic species from the *B. tabaci* complex colonize cassava and neighboring crops, but the feeding interactions between the different crops and *B. tabaci* species are unknown. The electrical penetration graph (EPG) technique makes it possible to conduct detailed feeding studies of sap-sucking insects by creating an electric circuit through the insect and the plant. The apparatus measures the voltage fluctuations while the wired-up insect feeds and produces graphs that describe feeding behavior. We utilized EPG to explore the feeding behavior of cassava-colonizing whiteflies (SSA1-SG3) on cassava, sweet potato, tomato, and cotton; and sweet potato-colonizing whiteflies (MED and IO) on cassava and sweet potato. Results show that: (1) feeding of SSA1-SG3 is not restricted to cassava. The least preferred host for SSA1-SG3 was tomato, where probing was delayed by 99 min compared to 10 min on other hosts, furthermore mean duration of phloem ingestion events was 36 min compared to 260 min on cassava. (2) Feeding of MED on cassava appeared to be non-functional, as it was characterized by short total phloem ingestion periods (<1 h) and few, short ingestion events, in contrast to feeding on sweet potato which was characterized by long phloem ingestion periods (>5 h). (3) Wire diameter affects the feeding in a statistically and practically significant manner. Implications for whitefly control and studies of host whitefly resistance are discussed.

## Introduction

Cassava (*Manihot esculenta* Crantz) is a semi-perennial shrub from the family Euphorbiaceae. It is primarily grown for its starchy tuberous roots, although the leaves are also used as a vegetable. African cassava production of more than 14 million tons is greater than production of the rest of the world combined, and cassava represents the staple food in many African countries (FAOSTAT, 2017). Cassava's resistance to drought, poor soils, increased CO_2_ levels and changing climatic conditions combined with its semi-perennial nature make it a resilient crop that farmers can rely on. It is grown by millions of smallholder farmers for subsistence and cash together with other crops such as sweet potato (*Ipomoea batatas* L.). Sweet potato is commonly grown in close association with cassava, and some studies even point out the benefits of intercropping the two (Legg et al., 2015).

The production of cassava in Africa is threatened by two viral diseases, cassava mosaic disease (CMD), caused by nine cassava mosaic begomoviruses (CMBs), and cassava brown streak disease (CBSD), caused by two cassava brown streak ipomoviruses (CBSIs) (Legg et al., 2015). Severe CMD became a major regional problem after the epidemic in the 1990s in Uganda and causes yield losses of more than 45 million tons every year (Thresh et al., 1994; Legg et al., 2014a,b). Successes in developing and disseminating varieties with strong resistance to CMD helped to bring that disease under control, although it still limits cassava production. In 2004 an outbreak of CBSD was reported in Uganda (Alicai et al., 2007). Its severity comes from the brown, corky, necrotic lesions it causes in the roots, rendering them unusable (Nichols, 1950). Today, CBSD still causes widespread damage across East, Central and Southern African countries. In addition, there is a great risk of spread to West Africa, the biggest cassava producing region in Africa, where a CBSD epidemic would have devastating consequences on food security (Legg et al., 2014a,b).

*Bemisia tabaci* (Gennadius) (Hemiptera, Aleyrodidae) is by far the most common whitefly on cassava and is the vector of CMBs and CBSIs (Legg et al., 2014a; Rey and Vanderschuren, 2017). Years of research concluded that *B. tabaci* and changes in its populations play the main role in driving the outbreaks and spread of both CMD and CBSD. The virus outbreaks have resulted from the rapid expansion of “superabundant” whitefly populations leading to fast and efficient virus transmission (Legg and Ogwal, 1998; Legg et al., 2011, 2014a).

*Bemisia tabaci* is a cryptic species complex consisting of many morphologically indistinguishable species and is recognized as a global pest (De Barro et al., 2011). The most commonly used taxonomic character is a segment of the mitochondrial gene for cytochrome oxidase I (mtCOI) which divides the species complex into at least 34 major species (Boykin et al., 2012; Boykin and De Barro, 2014). Cryptic *B. tabaci* species are therefore simply referred to as species through this study. However, recent studies using more comprehensive techniques, such as genome-wide single nucleotide polymorphism (SNP) markers, indicate that mtCOI is not the best taxonomic marker as it does not accurately represent the genetic differences between populations of cassava-colonizing whiteflies (Wosula et al., 2017).

*Bemisia tabaci* whiteflies in sub-Saharan Africa fall into two major monophyletic clades, the cassava-colonizing whiteflies, and those colonizing other crops—mostly vegetables. Five groups of cassava-colonizing *B. tabaci* have been identified in sub-Saharan Africa based on mtCOI and named SSA1-5, with SSA1 being further divided into five subgroups (SSA1-SG1 to SSA1-SG5) (Berry et al., 2004; Esterhuizen et al., 2013; Legg et al., 2014c; Ghosh et al., 2015). Population genetics studies revealed that SSA1-SG1, most of which fall within the new genetic group SSA-ECA (sub-Saharan Africa, East and Central Africa) based on SNPs, is the one associated with virus outbreaks (Tajebe et al., 2015a,b; Wosula et al., 2017). Vegetable-colonizing *B. tabaci* types in sub-Saharan Africa mostly belong to groups named Indian Ocean (IO), Mediterranean (MED, formerly Q), Middle East-Asia Minor 1 (MEAM1, formerly B or *Bemisia argentifolii*), and Uganda (Sseruwagi et al., 2005; Delatte et al., 2006; Boykin, 2014; Tocko-Marabena et al., 2017).

While *B. tabaci* is considered polyphagous, non-cassava *B. tabaci* are almost never found on cassava, and are not able to survive on cassava hosts for more than a couple of days in no-choice experiments (Legg, 1996). Moreover, the *B. tabaci* species (MEAM1, MED, and New World) occurring in South America have never been reported to colonize cassava (Carabalí et al., 2010a). It has been speculated that mortality on cassava of non-cassava species could be caused by cyanide poisoning since studies have shown the presence of bound cyanides in the phloem of cassava (Calatayud et al., 1994a,b). Cassava-colonizing *B. tabaci* species strongly prefer cassava, but there is evidence of their broader host range (Sseruwagi et al., 2006), especially in no-choice experiments. At this point, there is no answer to the question of why cassava can be colonized by some species of *B. tabaci* but not by others.

While there have been no feeding studies of cassava-colonizing whiteflies, it is known that feeding characteristics of the sap-sucking insects, including whiteflies, are directly correlated to host suitability and virus transmission efficiency (Jiang et al., 2000). A technique that enables detailed study of whitefly feeding in real-time is the Electrical Penetration Graph (EPG) system developed in the 1950s by McLean and Kinsey (1964). Today, EPG is a mature technique for studying sap-sucking insects with greatly improved design utilizing direct current electricity (DC) (McLean and Kinsey, 1964; Tjallingii, 1978; Backus et al., 2016). It is based on passing an electric current through soil and into a plant on which an insect tethered to a miniature electrode is placed to feed. When the insect inserts its stylet into the plant, a circuit is completed, current flows, and the pattern of voltage fluctuations can be measured and presented graphically on a computer screen (Walker, 2000). The graphical output gives a precise indication of the characteristics of the insect feeding (Janssen et al., 1989; Jiang et al., 1999). After the first studies established the EPG technique for whiteflies it has subsequently been used on *B. tabaci* in studies focusing on virus transmission, host resistance factors and insecticide effects (Walker and Perring, 1994; Jiang et al., 1999, 2000, 2001; Johnson and Walker, 1999; Jiang and Walker, 2001, 2003; Johnson et al., 2002; Rodríguez-López et al., 2011, 2012; Liu et al., 2012, 2013; Civolani et al., 2014; Zhou, 2014; Prado Maluta et al., 2017). To date there are no EPG studies of cassava-colonizing whitefly species. The only *B. tabaci* species studied using EPG are MEAM1 and MED. With respect to host plants, there is only one EPG study on cassava, which focused on the cassava mealybug (*Phenacoccus manihoti* Matile-Ferrero) (Calatayud et al., 1994b). Most EPG studies on whiteflies (10/15) were carried out using 12.5 μm gold wire, five times thicker than the 2.5 μm platinum wire used in other studies. The effect of this method was examined by Walker and Perring (1994) and they showed that thicker wire significantly impairs the mobility of whiteflies, although this effect was not quantified. Surprisingly, all the studies with 12.5 μm wire were done after 1994, which was when Walker and Perring pointed out the drawbacks.

The study reported here utilized the EPG technique to understand how different *B. tabaci* species (SSA1-SG3, MED, IO) feed on cassava and sweet potato, and how cassava-colonizing SSA1-SG3 feeds on non-hosts (sweet potato, cotton, and tomato). It aimed to provide insights into whitefly host acceptance or rejection, and to set the baseline for detailed cassava virus transmission characterization, an area that remains little explored by research decades after the initial devastating virus epidemics. Finally, we aimed to refine the EPG technique by quantifying the benefits of using 2.5 μm platinum wire over the thicker 12.5 μm gold wire.

## Materials and Methods

### Plant Material

All plants used in the study were grown under greenhouse conditions at the International Institute of Tropical Agriculture (IITA), Dar es Salaam, Tanzania. The varieties of cassava and tomato used were Albert and Moneymaker, respectively, while sweet potato and cotton were local landraces. All greenhouse plants were grown in pots with a soil mix of forest soil and manure mixed in a 4:1 ratio. Plants were typically 20–30 cm tall or with a minimum of five leaves. “Albert” is known to be preferred by whiteflies, while the tomato cultivar—Moneymaker—is extensively used in the scientific literature.

Considering the numerous reports of vector feeding behavior manipulation by viruses, it was important to ensure the use of virus-free cassava plants (Liu et al., 2013; Moreno-Delafuente et al., 2013; Lu et al., 2017). All cassava planting material was obtained from CMD and CBSD asymptomatic fields in Mtwara Region, Tanzania. Furthermore, leaf samples were taken from each stem and tested for the presence of Cassava Brown Streak Ipomoviruses using real-time RT-PCR to exclude the possibility of asymptomatic infections. The CBSI virus testing was done using the protocol described for cassava by Shirima et al. (2017). Leaf samples in the form of the middle leaflet were taken from the fifth youngest leaf from each cassava stem cutting used for planting. Samples were dried between two sheets of paper at room temperature for 4 days. Total RNA was extracted using the acetyltrimetyl ammonium bromide (CTAB) protocol, cassava complementary DNA (cDNA) was synthetized and real-time polymerase chain reaction was performed using primers, probes, and cycling conditions described in Shirima et al. (2017). “Albert” is resistant to CMD, and since only symptomless plants were selected for planting material, the risk of infection was considered low and the material was not tested for the presence of CMBs. The assumption of the absence CMD was further supported as none of the grown plants exhibited the symptoms. Sweet potato plants were asymptomatic. Vegetative material used to plant them has been maintained under insect-proof screenhouse conditions, without symptoms of virus infection, for 3 years. Tomato and cotton were grown from certified seed.

### Whitefly Colonies

Three species of whiteflies (*B. tabaci*) were used in in this study: (1) SSA1-SG3 which colonizes cassava in coastal Tanzania, (2) Indian Ocean (IO), and (3) MED, both of which colonize sweet potato and other vegetables, but not cassava. The SSA1-SG3 colony was established by collecting whiteflies from cassava at Chambezi in Bagamoyo District, Coast Region in May 2017. The IO and MED colonies were established by collecting whiteflies from sweet potato at Kibaha Research Station, Kibaha District, Coast Region in June 2017. The colonies were maintained in the greenhouse with partially controlled environment conditions (installed with cooling fans and air conditioning) with natural light (12L: 12D) and temperature ranging between 24 and 35°C. The colonies were reared on their respective host plants: SSA1-SG3 on cassava variety Albert; IO and MED on sweet potato. Host plants were planted in 30 cm diameter plastic pots held in meshed cages (100 × 50 × 50 cm). The colonies were transferred to fresh plants at intervals of 3–4 weeks. The colony species were periodically confirmed by PCR and partial sequencing of the mitochondrial DNA cytochrome oxidase I (mtCOI) gene.

### Electrical Penetration Graph (EPG)

EPG experiments were performed using a direct current Giga-8d DC-EPG device with 1 Giga-ohm input resistance capable of recording eight insects at a time (EPG systems, Wageningen, The Netherlands). Plants and the EPG equipment were placed in a faraday cage (1.5 × 1.5 × 1.5 m) to shield the setup from electrical noise. The voltage sampling rate was the default of 100 Hz. At the beginning of the recording, the graph was monitored and the substrate voltage was set to the required positive value for each channel to scale the signal to −5 to +5 volts and achieve the maximum resolution. In the case of a weak signal that could not be adjusted by increasing the substrate voltage, the gain on the EPG Giga-8d device was adjusted between the default of 50X and 100X. Data were recorded using the EPG Systems Stylet+d data acquisition software, v01.28 (04-05-2016)/B28. The whole EPG setup was housed in a windowless room with constant artificial plant light (T5 Fluorescent light, Maxibright, Chesterfield, UK), temperature of 26°C, and relative humidity of 60%. Plants were not subjected to drought stress and were always watered 1 h before the EPG recording to ensure the electrical conductivity of the soil during the 12 h recording time.

### Wiring of Whiteflies

Insects were carefully collected from the colonies in a glass vial by aspiration. Only female whiteflies were used to exclude the potential effect of sex on feeding. Additionally, female *B. tabaci* are more efficient in virus transmission and therefore preferred in previous whitefly studies (Costa and Bennett, 1950; Cohen and Nitzany, 1966). Before wiring, the glass vial was rolled on ice for 5–10 s to reduce the mobility of the whiteflies. Immediately after, whiteflies were placed on the ice-cold petri dish and quickly wired. Each female adult was glued to a 1 cm long, 2.5 μm thick platinum wire (Sigmund Cohn Corp, Mt Vernon, NY, USA), previously dipped in conductive water-based silver glue (EPG Systems, Wageningen, The Netherlands). The other end of the platinum wire was previously attached to a 2.5 cm long copper wire, which was itself soldered to a brass nail that inserts into the probe of the EPG device. The silver coating of the platinum wire was removed using 40% nitric acid after first gluing the wire to the copper wire. For the comparative study on the effects of wire thickness, 12.5 μm thick and 2 cm long gold wires (EPG Systems, Wageningen, The Netherlands) were used in the same brass nail-copper wire configuration. Once wired, each attached insect was lifted and set to rest on a cardboard surface until the remaining insects were wired. The wiring of 10 insects (eight required and two extra) was completed in ~20 min. After 5 min, wired insects were connected to the EPG probe for recording.

### Experimental Setup

One insect was placed on one plant, and plants were used only once. Insects were placed on the abaxial surface of one of the top three leaves of the plant. To access the abaxial surface, leaves were inverted, and the base of the leaf was taped to a solid surface. Eight plants were recorded at one time for 12 h. Four of the eight were always cassava, and the other four were either sweet potato, cotton, or tomato allowing the comparisons between the sets to determine if external factors affected the sets differently. The feeding behavior of SSA1-SG3 was studied on cassava, sweet potato, cotton, and tomato to assess the potential broader host range on these crops that are often grown in close proximity. Due to the time and resource limitations, the feeding behavior of MED whiteflies was only tested on cassava and sweet potato. The number of SSA1-SG3 individuals (replicates) used for statistical analysis, after excluding poor quality recordings, was 40, 15, 15, and 14 on cassava, sweet potato, tomato, and cotton hosts, respectively. Number of MED individuals was 28 and 15 on cassava and sweet potato, respectively, while for IO it was 5 and 9 on cassava and sweet potato, respectively. This study however does not draw conclusions about IO whiteflies due to the lower number of individuals.

### Whitefly Identification

After the 12-h recording period, whiteflies were recovered from the wire for species identification. The DNA of each recovered whitefly was extracted using the protocol as described by Wosula et al. (2017) with slight modifications. Whiteflies preserved in ethanol were put in the petri dish under the microscope and ethanol was removed by decanting and drying. Dry whiteflies were then picked and placed into a 1.5 ml tube containing 3 μl of lysis buffer (10 mM Tris-HCl (pH 8.0), 50 mM KCl, 2.5 mM MgCl_2_, 0.45% Tween-20, 0.01% Gelatine, 60 μg/ml Proteinase K). Whiteflies were then thoroughly ground using a 10 μl pipette tip. After grinding, an additional 20 μl of lysis buffer was added and the tube was vortexed and spun down. The tube was then incubated for 30 min at 55°C in a water bath, which was at the optimal temperature for Proteinase K digestion. Incubated samples were vortexed, spun down, and diluted to a 1:10 ratio in sterile distilled water. Diluted extract was used directly as a DNA template in PCR.

A 859 bp fragment of the mitochondrial gene for Cytochrome Oxidase I (mtCOI) was amplified using PCR with the BTSM-2-F (5′ TCTGGTTYTTTGGTCATCC 3′) and BTSM-3-R (5′ CACTTTCTGCCACATTAGA 3′) primer pair. The reaction contained 1X QuickLoad Master Mix (New England Biolabs, UK), 1 mM MgCl_2_, 0.24 μM of each primer, 2 μl of diluted DNA extract, and sterile distilled water to achieve the desired reaction volume of 25 μl. The thermal profile consisted of an initial denaturation step for 5 min at 95°C, 35 cycles of denaturation for 40 s at 94°C, annealing for 30 s at 52°C, and extension for 50 s at 72°C, and final extension for 10 min at 72°C. DNA electrophoresis was performed to confirm the successful and specific amplification of the desired fragment. PCR products with Purple Loading Dye (New England Biolabs, Ipswich, MA, USA) were loaded on to a 1% agarose gel stained with GelRed™ (Biotium, Fremont, CA, USA) and run for 45 min at 5 V/cm. Upon confirmation of amplification, PCR products were sent to Macrogen Inc. (Maryland, USA) for purification and direct sequencing.

The ends of the raw Sanger reads of forward and reverse sequences were manually trimmed to remove the low quality and ambiguous bases using Ridom Trace Edit v1.1.0 software. Forward and reverse sequences were then assembled and aligned using CLC Main Workbench v7.9.1 (QIAGEN, Aarhus, Denmark) and trimmed to the same length. All unique sequences were identified using the BLASTn algorithm of GenBank (http://www.ncbi.nlm.nih.gov). Trimmed Clustal W alignment of sequences, top hit sequences from GenBank, and a *Bemisia afer* outgroup sequence were used to create a maximum likelihood phylogenetic tree with 1,000 bootstrap replicates using MEGA (version 7.0), and whitefly species were identified.

### EPG Data Analysis

Raw EPG data recorded by EPG Systems Stylet+d was manually annotated using EPG Systems Stylet+a software v01.30 (13-04-2016)/B27. Annotated waveforms were non-probing (np), pathway (C), phloem salivation (E1), phloem ingestion (E2), derailed stylet mechanics (F), xylem feeding (G) and intracellular puncture—potential drop (pd). Waveforms were identified based on the waveform pattern, amplitude, relative voltage level, R/emf origin, frequency, and the context of the waveform as described in the previous EPG studies of *B. tabaci* (Jiang et al., 1999; Johnson and Walker, 1999; Liu et al., 2012; Civolani et al., 2014; Zhou, 2014; Prado Maluta et al., 2017).

Annotation files were then directly passed to a modified version of the Ebert 3.0 program in SAS Enterprise Guide 7.1, SAS 9.4 statistical software (SAS Institute, Cary, NC, USA) for further analysis which produces the same parameters as the popular Sarria excel workbook (Sarria et al., 2009; Ebert et al., 2015). The modified version is provided in the Supplementary Material. The modified version utilizes the series of BoxCox power transformation to determine the best possible transformation, as implemented in the PROC TRANSREG statement (Osborne, 2010). The results of this power transformation were inspected visually using histogram and Q-Q plots. For certain parameters, power transformations are unsuitable as they cannot approximate the necessary S-curve. Therefore, the Arcsine transformation was applied before the BoxCox transformation if this was necessary. The modification of the original Ebert 3.0 program also utilizes a macro script developed by Piepho (2012) that mitigates the case when varying standard error of a difference causes the traditional algorithm to fail to represent all significant differences of the means using the letter grouping. The Piepho algorithm solves the problem as it is able to generate a discontinous line display (Piepho, 2012). As a consequence, seeing discontinous assigned letters such as “ac” or “acd” is not uncommon (Piepho, 2014; Poosapati et al., 2014; Santos et al., 2015; McCaghey et al., 2017).

## Results

### Data Quality

Out of 240 wired whiteflies, 180 (75%) were successfully recorded for 12 h. The remaining 25% detached during mounting on the probe or escaped before the 12 h period elapsed. After removing poor quality recordings, 146/180 (81%) high quality recordings remained. Recording was considered as “bad” in the case of too high noise or too weak signal to identify the waveforms, interruption due to the leaf detachment or irregular feeding due to a damaged whitefly. Out of 146 successful recordings, 8 whiteflies were not recovered since they escaped after the 12 h recording but before the recovery.

Whiteflies attached to the 12.5 μm gold wire were not recovered for identification and are not included in the previous data quality numbers. Their identity is assumed to be SSA1-SG3 since they came from the pure colony on cassava. The thicker gold wire allowed successful 12-h recording of 22/24 (92%) wired whiteflies, the escape rates were extremely low. It was notable that with the gold wire, whitefly mortality was occasionally observed ~20 h after initiating EPG, which was never observed with the platinum wire.

### Whitefly Identification

**Figure 1:**
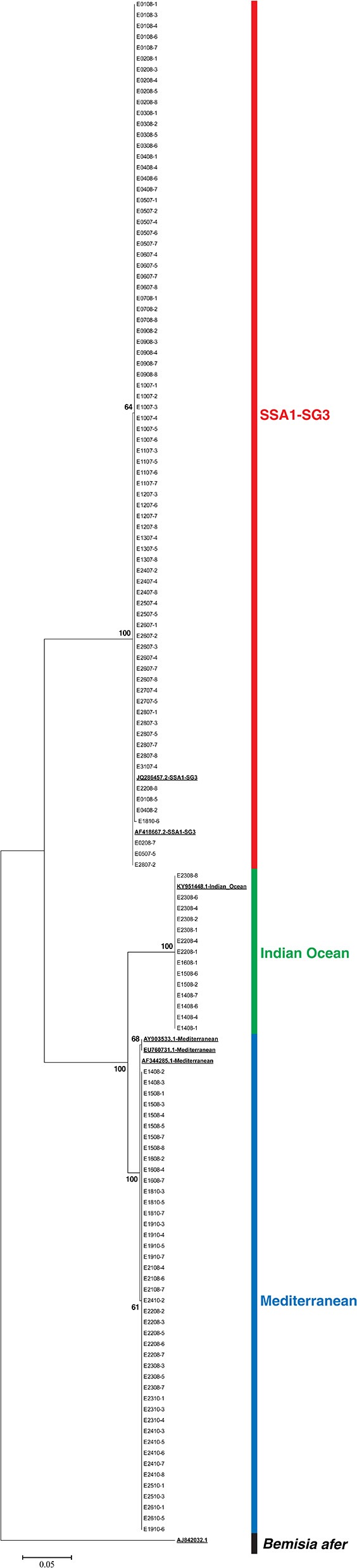
Maximum likelihood phylogenetic tree of EPG recorded *Bemisia tabaci* whiteflies, reference sequences (underlined and bold), and *Bemisia afer* sequence as an outgroup.

DNA of all 138 recovered whiteflies was extracted and the expected 859 bp segment of mtCOI was amplified. Good sequences were obtained for 135 whiteflies. Four unique sequences were present in the initial alignment. After a BLAST search in GenBank, three had 100% identity with GenBank sequences JQ286457 (SSA1-SG3), AF418667 (SSA1-SG3), and KY951448 (IO) and one sequence had the “top hits” with 99% identity with sequences AY903533, EU760731, and AF344285 (all three corresponding to MED). These sequences were included as references in the data set for phylogenetic analysis. The standardized length after trimming sequences was 522 bp which was used for construction of a maximum likelihood phylogenetic tree (Figure 1). The sequences are provided in the FASTA file available in the Supplementary Materials of this article. Identification of species was performed based on the topology of the phylogenetic tree and the positions in the tree of test sequences relative to the reference sequences obtained from GenBank. All 76 whiteflies from the colony on cassava were identified as *B. tabaci* SSA1-SG3, 73 had 100% identity with sequence JQ286457, and 3 had 1 bp difference and 100% match with AF418667. Since the cassava colony was pure, and contamination was never recorded on cassava colonies, 8 additional whiteflies with EPG data from cassava that were not successfully recovered were included in the study. Whiteflies from the sweet potato colony were identified as follows: 14 IO, 43 MED from East Africa, and 2 as SSA1-SG3. The two individuals that belonged to SSA1-SG3 were excluded from the study. Three whiteflies from the sweet potato colony without good sequences but with good EPG were excluded from the study.

### Cassava Virus Testing

Out of 60 cassava cuttings obtained from the field, one tested positive for UCBSV and was not planted. None were positive to CBSV. The technique was verified by positive detection on UCBSV and CBSV control samples, and no detection in the negative control.

### Epg Interpretation, Parameters, and Statistical Analysis

**Figure 2:**
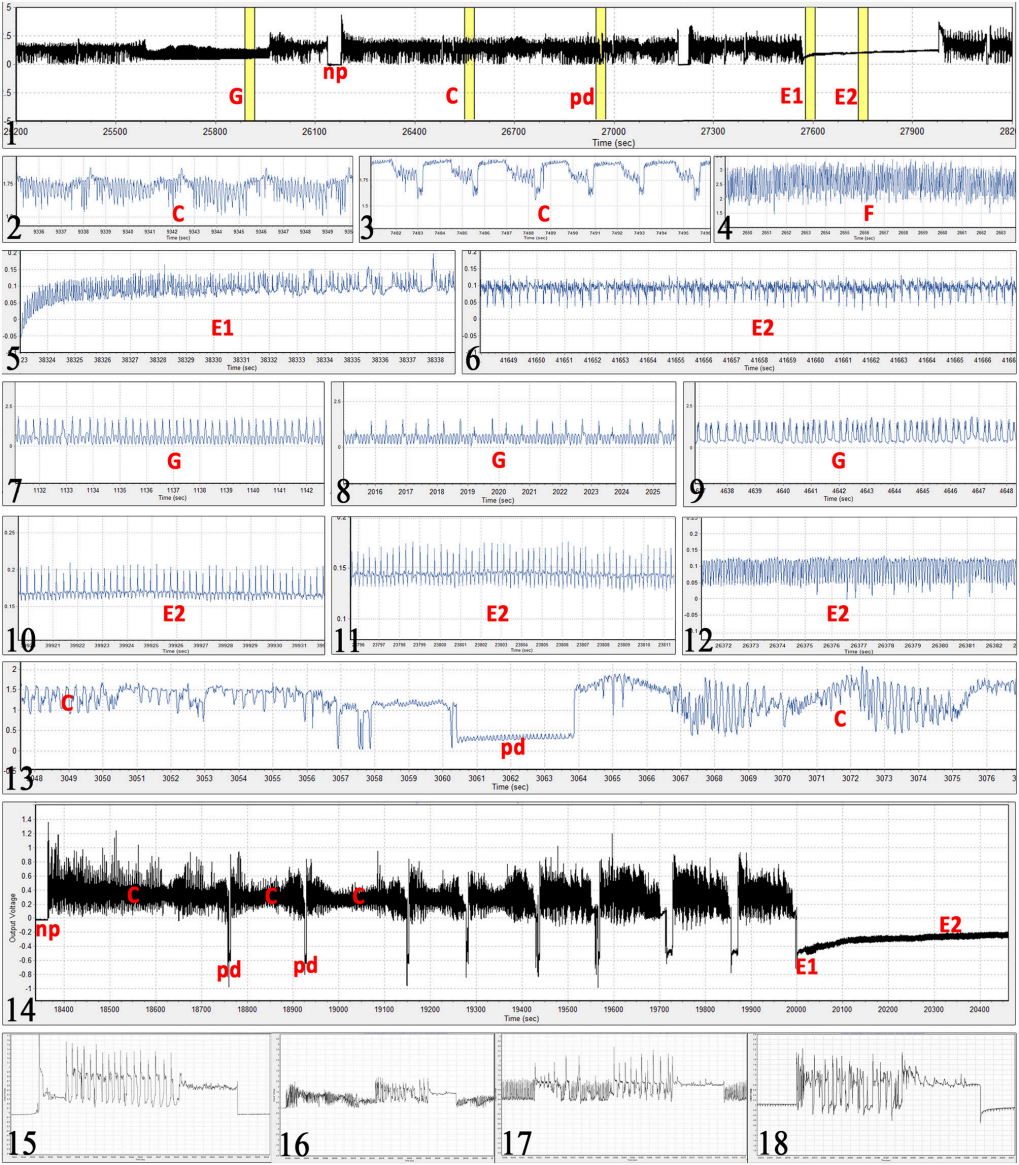
*Bemisia tabaci* EPG waveforms observed in the study. (Figure 1) Example of 1 h EPG with xylem feeding (G), non-probing (np), stylet pathway (C), potential drop (pd), phloem salivation (E1), and phloem ingestion (E2) waveforms. (2) stylet pathway and sheath salivation (C). (3) different form of the pathway waveform C. (4) derailed stylet mechanics (F), note the high frequency. (5) phloem salivation (E1) from its beginning, notice gradual increase in voltage level. (6) typical passive phloem ingestion (E2). (7–9) different forms of xylem feeding waveform (G), note that this waveform even though similar to those in Figures 5, 10, and 11, occurs at extracellular voltage levels (does not start with a potential drop). (10–12) different forms of phloem ingestion (E2). (13) intracellular puncture (pd) during pathway (C). (14) example of first 35 min of a probe with 8 potential drops (pd) before reaching the sieve element of the phloem (E1 followed by E2). (15–18) oviposition waveform during non-probing (np), stylet pathway (C),) xylem feeding (G), and phloem ingestion (E2), respectively. Ticks on the time axis are 1 s for all graphs except for: graph (1) 5 min; (14) 100 s; (16) 2 s. Absolute voltage values on the y-axis are arbitrary in the EPG recordings.

**Figure 3:**
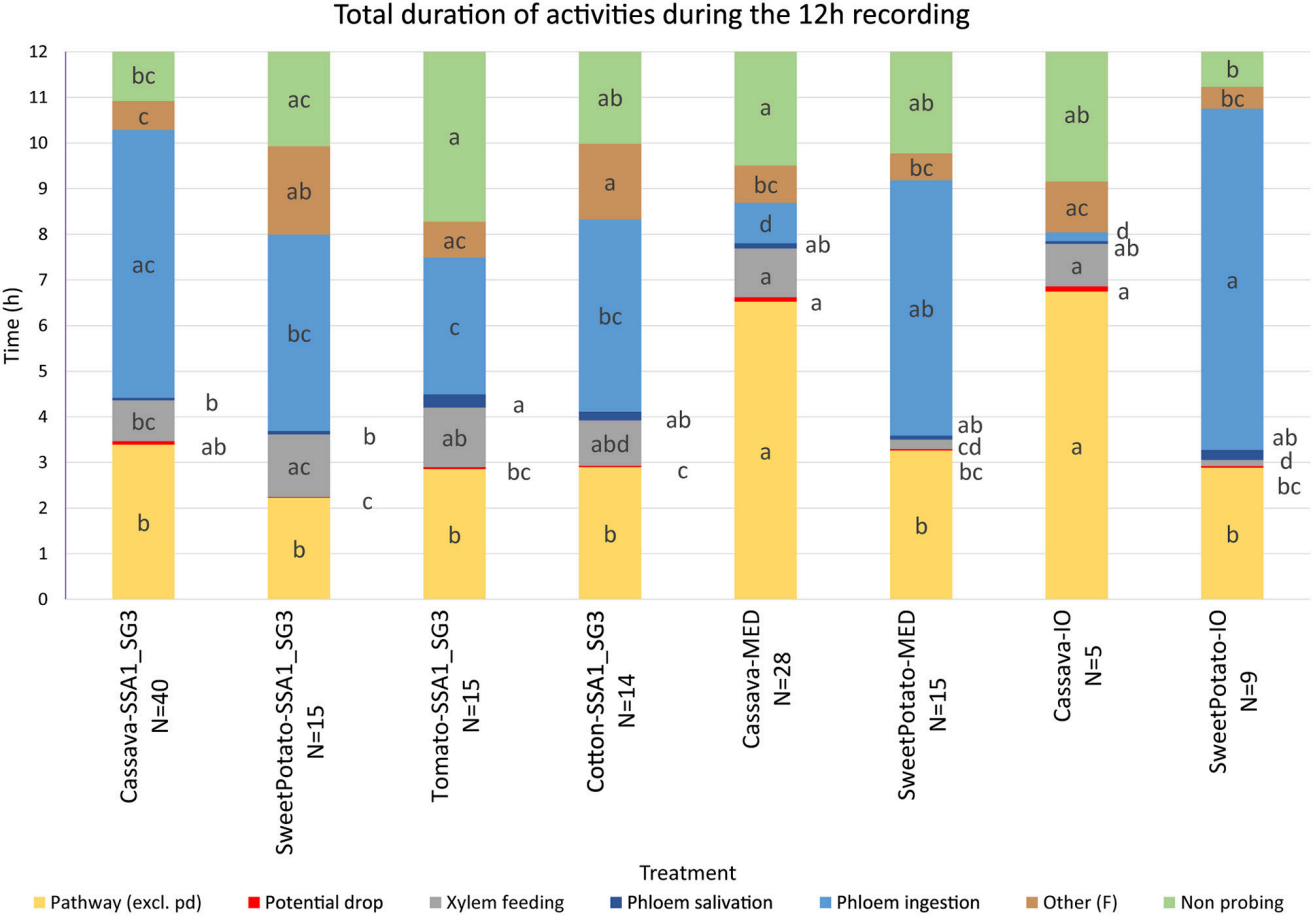
Total duration of activities during the 12-h recording period. Statistical analysis was performed after transformation, data presented is on the original scale. Letter display was obtained by the method of Piepho (2012) from PROC GLIMMIX output. Values with the same letter are not significantly different (*P* = 0.05, Tukey-Kramer test). The number of SSA1-SG3 individuals was 40, 15, 15, and 14 on cassava, sweet potato, tomato, and cotton hosts, respectively, 28 and 15 of MED on cassava and sweet potato, respectively, and 5 and 9 of IO individuals on cassava and sweet potato, respectively.

Observed waveforms in the EPG recordings were consistent with those previously described in the literature on *B. tabaci* (Janssen et al., 1989; Jiang et al., 1999; Johnson and Walker, 1999; Liu et al., 2012; Zhou, 2014) and are presented in Figure 2. Each phloem ingestion (E2) event was preceded by a phloem salivation waveform (E1). Interestingly, different E2 waveform variants as described by Zhou (2014) were also occasionally observed in this study. In this study, waveform F (derailed stylet mechanics) also included the waveform with the pattern similar to xylem ingestion waveform G but occurring <10 min from the beginning of the probe. It is assumed that whiteflies are unable to reach xylem in <10 min (Prof. Gregory P. Walker, University of California Riverside, personal communication). The statistical analysis was performed on transformed data, and tabular data presented here are on the original scale for intuitive understanding of the values. Transformed data, confidence intervals on the transformed scale, BoxCox transformation exponents and Arcsine transformed values (where applied) are available for all EPG parameters in the Supplementary Material (Supplementary Table S1). The analysis of the data from SSA1-SG3 whiteflies on cassava from three different sets confirmed no significant differences and were combined for the final analysis. Comparison between the MED and IO showed no significant differences. However, since the number of individuals in the IO set was low, this study focuses and draws conclusions solely from MED. The basic EPG parameters for IO are shown in Figure 3, and Tables 1, 2.

**Table 1:** Mean durations of individual waveform events.

	**Mean duration of individual waveform events (min, pd and E1 in seconds)**						
	**C**	**pd**	**G**	**E1**	**E2**	**F**	**np**
Cassava-SSA1-SG3	10.3 a	6.0 a	37.0 ab	65.6 b	260 a	19.3 ab	5.0 b
SweetPotato-SSA1-SG3	3.8 d	4.3 bc	61.1 ab	89.5 ab	145.1 a	20.1 a	3.4 b
Tomato-SSA1-SG3	5.6 c	4.4 bc	57.6 a	91.1 a	35.8 bc	23.3 a	20.2 a
Cotton-SSA1-SG3	6.9 bc	5.1 ab	39.7 ab	92.3 ab	125.6 ab	23.1 a	5.3 abc
Cassava-MED	8.9 ab	5.0 b	15.2 c	96.9 ab	41.0 cd	10.9 bc	5.2 ab
SweetPotato-MED	6.1 c	3.8 cd	19.8 bc	60.3 ab	127.4 a	14.0 ac	5.3 abc
Cassava-IO	8.0 ac	5.6 abc	15.4 bc	104.8 ab	10.6 d	12.7 ac	3.6 abc
SweetPotato-IO	6.2 bcd	3.3 d	24.2 b	71.0 ab	130.7 ab	6.5 c	1.9 c

*Statistical analysis was performed after transformation, data presented is on the original scale. Letter display was obtained by the method of Piepho (2012) from PROC GLIMMIX output. Means with same letters within column are not significantly different (P = 0.05, Tukey-Kramer test)*.

*C, pathway; pd, intracellular puncture (potential drop); G, xylem feeding; E1, phloem salivation; E2, phloem ingestion; F, derailed stylet mechanics; np, non-probing. Waveform pd and E1 are expressed in seconds, and all other values are in minutes. The number of SSA1-SG3 individuals was 40, 15, 15, and 14 on cassava, sweet potato, tomato, and cotton hosts, respectively, 28 and 15 of MED on cassava and sweet potato, respectively, and 5 and 9 of IO individuals on cassava and sweet potato, respectively*.

**Table 2:** Mean number of occurrence (frequency) of each waveform.

	**Mean number of waveform events (rounded)**						
	**C**	**pd**	**G**	**E1**	**E2**	**F**	**np**
Cassava-SSA1-SG3	20 d	45 ac	2 cd	4 c	2 c	2 c	16 c
SweetPotato-SSA1-SG3	38 ac	15 d	2 bc	2 c	2 bc	6 a	35 ab
Tomato-SSA1-SG3	31 c	34 bcd	1 c	10 b	6 a	2 bc	22 ac
Cotton-SSA1-SG3	28 cd	22 cd	1 cd	4 bc	4 ac	5 a	25 bc
Cassava-MED	48 ab	74 a	4 a	4 ac	2 c	4 ab	39 b
SweetPotato-MED	34 bc	34 bc	1 cd	5 bc	4 ab	3 ac	30 ab
Cassava-IO	52 a	78 ab	4 ab	3 bc	1 bc	4 ac	44 ab
SweetPotato-IO	34 acd	42 ac	0 d	9 ab	7 a	4 ac	26 bc

*Statistical analysis was performed after transformation, data presented is on the original scale. Letter display was obtained by the method of Piepho (2012) from PROC GLIMMIX output. Means with same letters within column are not significantly different (P = 0.05, Tukey-Kramer test)*.

*C, pathway; pd, intracellular puncture (potential drop); G, xylem feeding; E1, phloem salivation; E2, phloem ingestion; F, derailed stylet mechanics; np, non-probing. The number of SSA1-SG3 individuals was 40, 15, 15, and 14 on cassava, sweet potato, tomato, and cotton hosts, respectively, 28 and 15 of MED on cassava and sweet potato, respectively, and 5 and 9 of IO individuals on cassava and sweet potato, respectively*.

### Effect of Wire Thickness on the EPG Results

**Figure 4:**
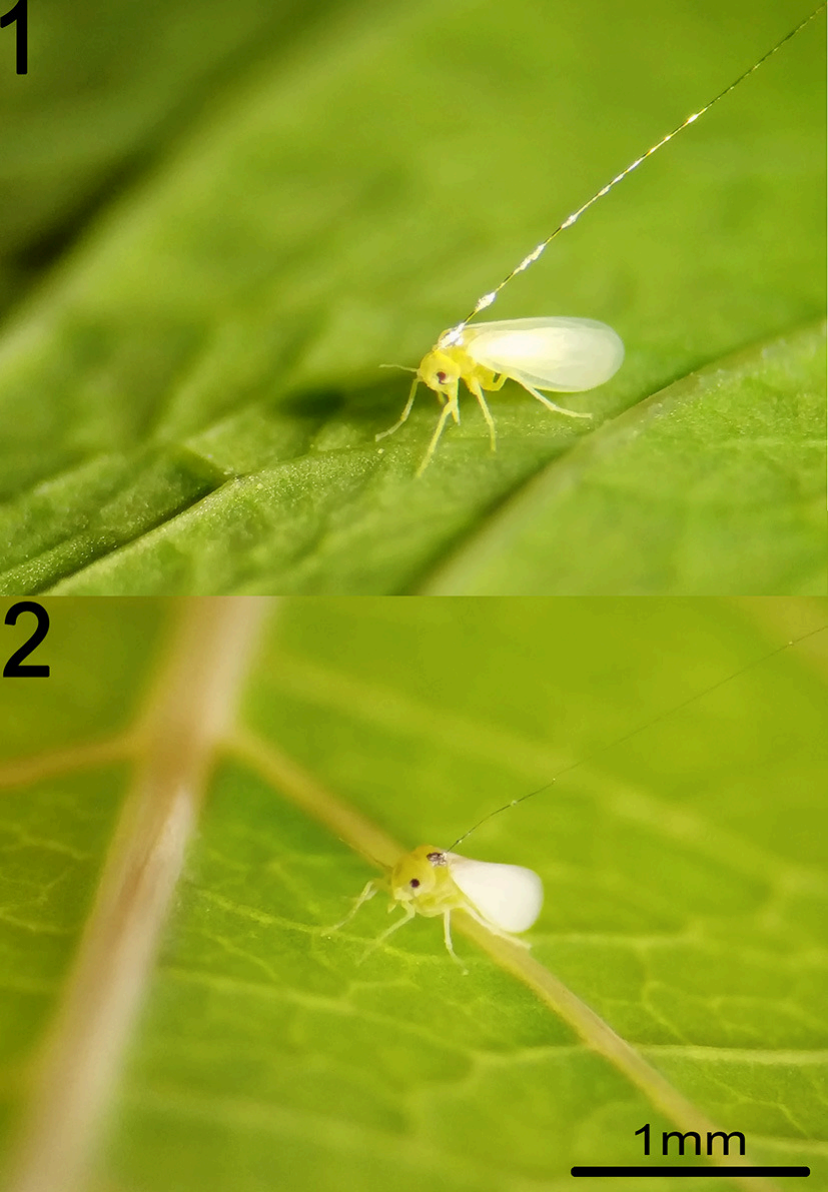
*Bemisia tabaci* SSA1-SG3 on the abaxial surface of a cassava leaf wired with 12.5 μm gold wire (1) and with 2.5 μm platinum wire (2).

An investigation of the effect of using 12.5 μm gold wire instead of the thinner 2.5 μm platinum wire on SSA1-SG3 whiteflies feeding on cassava showed large and statistically significant differences. Overall, 60% of the EPG parameters showed statistically significant differences between the different wire diameters. The main effect of the thicker wire can be described as causing more numerous shorter probes, making phloem access more difficult, drastically reducing time spent ingesting phloem sap, increasing the waveform F (derailed stylet mechanics), and causing less continuous feeding in general. Out of the most commonly used EPG parameters, only mean durations of potential drop (pd), mean duration of phloem salivation (E1), and mean and total duration of xylem feeding (G) were not affected by the thicker wire. Comparative visual observation of whiteflies tethered to different wires is shown in Figure 4 and restricted movement of whitefly is seen in the video available in the Supplementary Video. The comparison of the basic EPG parameters and a few more detailed parameters is shown in Table 3; the complete dataset is provided in the Supplementary Table S1.

**Table 3:** EPG parameters of SSA1-SG3 whiteflies feeding on cassava tethered to thin 2.5 μm platinum wire and thicker 12.5 μm gold wire.

	**2.5 μm platinum wire *N* = 40**	**12.5 μm gold wire *N* = 23**
Number of probes	16.3 b	76.2 a
Mean duration of probes in min	67.9 a	10.8 b
Total duration of C in min	203.3 b	276.1 a
Total duration of pd in min	4.5 a	2.4 b
Total duration of G in min	53.9 a	46.3 a
Total duration of E1 in min	3.4 a	1.4 b
Total duration of E2 in min	352 a	90.1 b
Total duration of F in min	38.3 b	95.2 a
Total duration of np in min	69.1 b	210.8 a
Mean duration of C in min	10.3 a	4.5 b
Mean duration of pd in min	0.1 a	0.1 a
Mean duration of G in min	37 a	56.1 a
Mean duration of E1 in min	1.1 a	0.7 a
Mean duration of E2 in min	260 a	64.2 b
Mean duration of F in min	19.3 a	18.2 a
Mean duration of np in min	5 a	4.1 b
Number of short C events	7.1 b	55 a
Duration of first probe in min	15.8 a	3.9 b
Duration of second probe in min	27.8 a	2.6 b
Number of F waveforms	1.7 b	5.5 a
Time from start of EPG to 1st E in min	196.5 b	304.9 a
Number of sustained E2	1.5 a	1 b
Duration of longest E2 in min	328.4 a	100.5 b
Time from start of probe with 1st E to 1st E in min	33.1 a	25.2 a
Percent of whiteflies with E2 waveform	95	64

*Statistical analysis was performed after transformation, data presented is on the original scale. Letter display was obtained by the method of Piepho (2012) from PROC GLIMMIX output. Means with same letters within row are not significantly different (P = 0.05, Tukey-Kramer test)*.

*C, pathway; pd, intracellular puncture (potential drop); G, xylem feeding; E1, phloem salivation; E2, phloem ingestion; E, any phloem event; F, derailed stylet mechanics; np, non-probing. The number of individuals was 40 and 23 for 2.5 μm and 12.5 μm wire, respectively*.

### SSA1-SG3 Whiteflies on Different Hosts

An analysis of the total duration of feeding components is presented in Figure 3, while mean durations and number of individual events are presented in Tables 1, 2, respectively. Cassava-colonizing SSA1-SG3 had significantly longer (10.3 min) mean pathway event duration (C) on cassava compared to other hosts (Table 1) although total durations did not differ (Figure 3). The mean duration of potential drops (pd) was significantly longer on cassava than on sweet potato and tomato, but not different from cotton (Table 1). Interestingly, the number of potential drops (pd) was much lower on sweet potato than on cassava (Table 2). The total duration of phloem salivation (E1) was significantly shorter on cassava and sweet potato, and longer on tomato, while on cotton it was in between (Figure 3). Longer total phloem salivation (E1) in tomato (Figure 3) resulted from significantly longer, and more frequent salivation events (Tables 1, 2). Total duration of phloem ingestion (E2) differed between plant hosts, with the longest phloem feeding on cassava, the shortest on tomato, and intermediate periods on sweet potato and cotton (Figure 3). The mean duration of phloem ingestion (E2) was highest on cassava (260 min), and significantly shorter on tomato lasting only 35.8 min (Table 1). Sweet potato and cotton fell in between, similarly to the other parameters. Total and mean non-probing time (np) (Figure 3 and Table 1, respectively) was significantly shorter on the natural host, cassava. In tomato, non-probe events (np) were four times longer (Table 1). Xylem feeding (G) characteristics were not different among the hosts (Table 1 and Figure 3). Total duration of the derailed stylet mechanics waveform (F) was longer on sweet potato and cotton than on cassava (Figure 3), due to the higher frequency of events (Table 2).

In less-preferred tomato, phloem salivation (E1) contributed 10.6% of the phloem phase, significantly more than on cassava, sweet potato and cotton which had 2.6–8.1% (Table 4). The first probe was delayed by 99.1 min, compared to under 10 min in other hosts. After the first probe has started, the time to reach the phloem was equal among all plant types. The time to phloem ingestion from the beginning of that probe was shortest on tomato, while cotton and sweet potato did not differ from cassava. A similar result was seen in the time to reach the phloem from the beginning of the first probe with phloem salivation. Whiteflies salivated significantly longer before achieving the first sustained phloem ingestion when feeding on tomato.

**Table 4:** Selected detailed EPG parameters describing whitefly-plant interactions.

	**Cassava SSA1-SG3**	**Sweet potato SSA1-SG3**	**Tomato SSA1-SG3**	**Cotton SSA1-SG3**	**Cassava MED**	**Sweet potato MED**
						
Percent contribution of E1 to phloem phase	8.1 b	2.6 b	10.6 a	5.9 b	48.5 a	1.7 b
Time in min from beginning of EPG to first probe	8.2 b	8.9 abc	99.1 a	3.2 bc	15.2 ab	1.3 c
Time in h from first probe to 1st E	3.1 a	4.9 a	2.9 a	3.9 a	4.2 a	2.6 a
Time in min to first E2 from start of that probe	37.6 a	30.1 ab	20.6 b	32.8 ab	24.3 b	27.2 ab
Time in min from start of probe with 1st E to 1st E	33.1 ab	28.4 a	17 b	32.3 ab	27.1 ab	25.1 ab
Duration in min of E1 followed by first sustained E2	0.7 b	1.7 a	2.8 a	1.4 ab	1.5 a	1 a
Number of sustained E2	1.5 b	2.1 bc	3.5 c	1.9 bc	0.8 a	3.1 c
Number of sustained G	1.2 ab	1.5 a	1.3 a	1.1 ab	1.8 a	0.4 b

*Statistical analysis was performed after transformation, data presented is on the original scale. Letter display was obtained by the method of Piepho (2012) from PROC GLIMMIX output. Means with same letters within row are not significantly different (P = 0.05, Tukey-Kramer test)*.

*G, xylem feeding; E1, phloem salivation; E2, phloem ingestion; E, any phloem event. The number of SSA1-SG3 individuals was 40, 15, 15, and 14 on cassava, sweet potato, tomato, and cotton hosts, respectively, 28 and 15 of MED on cassava and sweet potato, respectively*.

### SSA1-SG3 Whiteflies From Cassava vs. MED From Sweet Potato

The comparison of the two *B. tabaci* whitefly species (SSA1-SG3 and MED) on the sweet potato host showed an almost indistinguishable feeding behavior. The only significant differences were the shorter mean duration of the individual pathway phase (C) events in SSA1-SG3 (Table 1), and the higher number of potential drops (pd) in MED (Table 2). The total durations were however indistinguishable (Figure 3). When compared on the cassava host, dramatic differences in feeding became apparent. More specifically, the feeding of MED whiteflies on cassava was very different and impaired compared to the feeding of both SSA1-SG3 on cassava and of MED on sweet potato.

When forced to feed on cassava, MED whiteflies spent the same amount of time with the stylets in the leaf tissue (i.e., probing) as they did on their preferred host (Figure 3). However, on cassava they spent twice the amount of time in the pathway phase (C) and had longer and more frequent pathway events (Figure 3). They had longer potential drops on cassava, and these were more frequent (Tables 1, 2, respectively). Xylem feeding events (G) were significantly more common (Table 2), contributing to the longer total xylem feeding (Figure 3), although the mean duration of xylem events was the same (Table 1). There were no differences in phloem salivation (E1) (Tables 1, 2 and Figure 3). On the contrary, the most obvious differences were seen in phloem ingestion (E2). During the 12-h recording period, MED whiteflies spent <1 h ingesting the cassava phloem sap (E2), while on sweet potato, their preferred host, it was longer than 5 h (Figure 3). Mean duration of the phloem ingestion events (E2) was also significantly lower on cassava (Table 1), as well as the number of those events (Table 2). There were no differences in total and mean duration, and number of derailed stylet mechanics waveform (F) and non-probing events (np) between the two hosts (Tables 1,2 and Figure 3).

In addition to much shorter, and less continuous phloem phase periods in MED whiteflies, phloem salivation (E1) comprised over 48.5% of the phloem phase, compared to 1.7% on the preferred host (Table 4). Furthermore, not all MED whiteflies had sustained (>10 min) phloem ingestion (E2) resulting in the mean number of 0.8 sustained phloem ingestion (E2) events in 12 h, while on sweet potato they had 3.1 (Table 4). Similar to SSA1-SG3 on tomato, MED on cassava had a significant, albeit less dramatic, 15.2 min delay in time to the beginning of the first probe (Table 4). There were no significant differences in the parameters describing the difficulties in reaching the phloem (Table 4). Interestingly, in addition to the longer total duration (Figure 3) and frequency of xylem feeding (Table 2), there were 1.8 sustained xylem feeding events on cassava compared to 0.4 on sweet potato (Table 4).

## Discussion

The superabundance phenomenon of cassava-colonizing whiteflies associated with the decades-long pandemics of cassava viruses is still poorly understood. There is a need to understand the factors behind the ability of cassava-colonizing *B. tabaci* to feed and reproduce on cassava. Since the most important means of whitefly-plant interaction and recognition is through probing and feeding, we utilized the EPG technique to investigate these interactions in great detail. The results presented here for the feeding characterization of cassava-colonizing *B. tabaci* represent the first baseline measurement of cassava whiteflies and provide results with a high level of confidence considering the relatively large number of insects monitored (*N* = 40).

### SSA1-SG3 Can Successfully Feed on Cotton, Sweet Potato, and Tomato

The comparison of SSA1-SG3 whiteflies feeding on cassava, sweet potato, cotton and tomato most notably shows that SSA1-SG3 from coastal East Africa can successfully feed on all of these crops. This supports the finding of Sseruwagi et al. (2006) in Uganda that cassava whiteflies are not restricted to cassava. Based on these two sets of evidence it appears clear that *B. tabaci* occurring on cassava in East Africa do not have a host range that is restricted by the ability to feed. The practical significance of this finding is apparent as attempts to control whiteflies on cassava through the use of cassava-free periods are likely to be of limited value, since these whiteflies can feed on other crops. The whiteflies are likely to survive, even though they will be clean of cassava viruses, as demonstrated in the cassava community phytosanitation study (Legg et al., 2017). Knowing this, special attention should be paid when designing cultural control methods for whiteflies on cassava.

Cassava-colonizing SSA1-SG3 whitefly host suitability, based on the maximum and most continuous phloem ingestion, and the minimum non-probing time, is in the order: cassava > sweet potato > cotton > tomato. The much longer times taken before making the first probe on tomato suggest a much lower host preference even before the leaf is penetrated. Tomato trichomes probably make finding a suitable feeding location more difficult, especially for tethered whiteflies. The trichome factor, however, cannot completely explain the observations. Once a suitable feeding spot was found, whiteflies reached the phloem faster than on cassava, which rules out the existence of major epidermis or mesophyll associated resistance factors similar to those reported in the Mi tomato line where impaired feeding was attributed to epidermis and/or mesophyll factors (Jiang et al., 2001). The longer salivation before sustained phloem feeding and longer mean salivation on tomato shows that phloem factors such as taste perception and host resistance are also in play. Further studies are needed to clarify the apparent lower preference of tomato compared to the other host plants.

Feeding behavior alone revealed small differences between cassava and sweet potato hosts. However, this may not necessarily indicate that colonization of both hosts would be the same, particularly since these results were obtained from no-choice experiments. In practice, cassava whiteflies are rarely found on other hosts (Legg, 1996; Sseruwagi et al., 2006; Tajebe et al., 2015a). Other factors besides the ability to feed are important when it comes to host preference and colonization and they can be a combination of sight, olfactory stimuli, nutritional characteristics of the phloem sap as well as specific toxic or attractive chemicals for certain whitefly types. Additionally, reproductive success may not be the same on different host plants.

### MED Whiteflies Probe Cassava, but Feeding Is Severely Limited

MED whiteflies from sweet potato barely fed on the cassava phloem even after 12 h. Previous research (Legg, 1996) as well as our own informal observations suggest that adults of sweet potato colonizing species do not survive for more than a few days on cassava plants.

Based on the results of the study reported here, it seems that either starvation or poisoning could be plausible causes of this mortality. Increased xylem feeding, an indicator of dehydration, suggests that whiteflies are not ingesting enough phloem sap (Spiller et al., 1990; Powell and Hardie, 2002). The fact that there was no difference in mesophyll resistance parameters excludes the presence of resistance factors in the mesophyll. Additionally, the slightly longer time from the beginning of EPG to the first probe suggests that there was only a slightly lower preference for cassava compared to the sweet potato based on olfactory perceptions, which is what might be expected from a non-host plant. The high contribution of salivation to the phloem phase, the low number of sustained ingestion events, and the extremely low mean and total durations of phloem ingestion clearly indicate an inability to feed on cassava phloem. If it was possible to identify the plant or insect-associated factors that give rise to this reduced feeding response, it might be possible to utilize this information to develop cassava varieties with resistance to cassava-colonizing whiteflies.

Given the heavily reduced feeding, the risk of non-cassava whiteflies adapting to cassava over time, (e.g., in an intercropping system where the plants are grown in very close proximity) seems very low. Previous studies showed that non-cassava whiteflies cannot directly adapt and reproduce on cassava, suggesting that there may be underlying genetic factors at play (Legg, 1996; Carabalí et al., 2010a). Interestingly, the gradual adaptation of Colombian MEAM1 from beans to cassava after being passaged through several intermediate hosts resulted in some adaptation success. This suggests that given a strong enough selection pressure, at least some non-cassava whiteflies might be able to adapt to cassava within a period of 15 generations or more (Carabali et al., 2005).

To further investigate the cause of mortality, future studies might use longer EPG recordings (48–72 h) or artificial diets containing bound cyanides. If cyanide poisoning is responsible for reduced feeding and mortality of non-cassava whitefly, the corollary is that cassava whiteflies must have detoxification mechanisms that enable them to feed on cassava as well as a broad range of other host plants (Sseruwagi et al., 2006). Under these circumstances, RNAi based approaches aimed at “knocking out” detoxification enzyme systems might represent a useful future line of research investigation.

### Use of 12.5 μm Gold Wire Significantly and Substantially Affects the EPG Data

The comparison of two wire thicknesses reported in the literature shows that the use of thicker (12.5 μm) wire introduces significant biases in EPG data. In our investigation, the observed feeding behavior with the thick wire was completely different to natural feeding, which calls into question the practical significance of conclusions drawn from studies that have used thick wire. The thicker wire does enable higher recording efficiency, is far easier to handle, and is significantly cheaper—tempting researchers to choose this method. The possible reason for the widespread use of the thick wire may be the misinterpretation of the study by Lei et al. (1997), which compared 20 and 10 μm wires, and concluded that 10 μm is better than 20 μm. However, it is likely that 10 μm wire would have a similar negative effect on whitefly behavior as the 12.5 μm evaluated here. In behavioral studies, which by nature have a lot of variation and are hard to do consistently, it is vital to minimize all possible sources of bias. Even thinner 1.25 μm wire has been evaluated by Walker and Perring (1994). However, the conclusion of this study was that whilst both 2.5 and 1.25 μm wires allowed good whitefly mobility, the thinner of the two broke more often. The use of 1.25 μm or thinner would also increase the number of insect escapes and slow down the experimental setup. Therefore, we recommend the use of 2.5 μm wire as a standard protocol for whiteflies to ensure the acquisition of data that represents the natural feeding behavior as closely as technically possible, and to make future studies comparable to each other.

These data for a whitefly-preferred cassava variety (Albert) will be of great value as a baseline when seeking to identify sources of whitefly resistance, and the comparison of whitefly feeding behavior on diverse cassava varieties and wild relatives will be an important next step for this area of research. The potential of such screening is illustrated in the study by Carabalí et al. (2010b) which identified antixenosis-based resistance to the whitefly *Aleurotrachelus socialis* Bondar in a cassava relative, *Manihot flabellifolia* Pohl. Current research efforts have so far failed to identify strong sources of resistance to whiteflies in existing cassava germplasm resources (Omongo et al., 2012). This suggests that non-conventional approaches to the development of resistance need to be explored. Fundamental research might therefore examine what “prevents” non-cassava whiteflies from feeding effectively on cassava. This knowledge could then be applied to the development of novel strategies to completely prevent cassava whiteflies from feeding on cassava. The results of this investigation, therefore, lay the foundation for future studies aimed at developing sustainable strategies to reduce the impact of cassava viruses vectored by cassava-colonizing *B. tabaci*. Success in achieving this goal will impact positively on the lives of millions of smallholder farmers in Africa whose livelihoods depend on the production and sale of healthy cassava crops.

Finally, this study pioneered the electropenetrography technique in Africa and the established EPG laboratory is well-positioned to expand these studies to other important sap-sucking insects. Notable examples are the banana aphid (*Pentalonia nigronervosa* Coquerel)—vector of banana bunchy top virus, and the mealybug vectors of cocoa swollen shoot virus, which like whiteflies transmit viruses that negatively impact the lives of hundreds of millions of people. EPG is one of many tools that offer the potential to bring solutions to these devastating crop diseases and thereby have a lasting positive impact on global food security.

## Author Contributions

MM and ENW conducted the experiments. MM analyzed data and wrote the manuscript. All authors conceived and designed the research and read, revised, and approved the manuscript.

### Conflict of Interest Statement

The authors declare that the research was conducted in the absence of any commercial or financial relationships that could be construed as a potential conflict of interest.
